# Genetic and epigenetic studies of *FOXP3* in asthma and allergy

**DOI:** 10.1186/s40733-015-0012-4

**Published:** 2015-10-20

**Authors:** Cintia Rodrigues Marques, Ryan Santos Costa, Gustavo Nunes de Oliveira Costa, Thiago Magalhães da Silva, Tatiane Oliveira Teixeira, Emília Maria Medeiros de Andrade, Alana A. Galvão, Valdirene Leão Carneiro, Camila Alexandrina Figueiredo

**Affiliations:** 1 Instituto de Ciências da Saúde, Universidade Federal da Bahia, Avenida Reitor Miguel Calmon, s/n, Canela, CEP – 40110-100 Salvador, Bahia Brazil; 2 Instituto de Saúde Coletiva, Universidade Federal da Bahia, Salvador, Brazil; 3 Departamento de Ciências Biológicas, Universidade Estadual do Sudoeste da Bahia, Jequié, Brazil; 4 Departamento de Ciências da Vida, Universidade do Estado da Bahia, Salvador, Brazil

## Abstract

Multiple factors interact to trigger allergic diseases, including individual genetic background and factors related to the environment such as exposure to allergens, air pollution and respiratory infections. The FOXP3 transcription factor is constitutively expressed in CD4^+^CD25^+^FOXP3^+^ regulatory T cells (Tregs) and is critical for the maintenance of immune homeostasis. For example, FOXP3 is responsible for the suppression of the Th2 response following exposure to allergens. Studies have shown that expression of the *FOXP3* gene is reduced in patients with asthma and allergies compared to healthy controls. Therefore, the impairment of FOXP3 function caused by genetic polymorphisms and/or epigenetic mechanisms may be involved in the etiology of asthma and other allergic diseases. This review discusses some aspects of the role of FOXP3 in the development of asthma and allergy, with a particular emphasis on genetic and epigenetic factors.

## Background

Allergic respiratory diseases such as asthma and rhinitis are considered a serious public health problem and have an increasing prevalence in all regions of the world, regardless of the economic and social development of these regions [1]. According to the World Health Organization (WHO), each year approximately 250,000 deaths are due to asthma. and the estimated number of individuals affected by this disease has reached 300 million people worldwide [2].

Despite the increasing technological advancements of molecular biology research and the substantial exploration of the genetics and epigenetics of asthma and other allergic diseases, the immune mechanisms of such diseases remain unclear. However, in recent years, these studies have raised new interest in the regulatory molecules of the immune system [3, 4]. Some researchers have hypothesized that the genetic variations and epigenetic changes that affect molecules found in regulatory T cells, such as the FOXP3 gene, can cause dysfunction of regulatory T cells and can thus influence the development of immune-mediated diseases. The present review aims to provide an overview of FOXP3 role in immune regulatory processes as well as a discussion of the implications of this activity on allergic diseases, specifically asthma and allergic rhinitis.

### FOXP3, Asthma and Allergies

Respiratory allergies are complex diseases that are triggered by multiple interacting factors, including an individual’s genetic background and factors related to the environment, such as allergen exposure, air pollution and respiratory infection [5, 6]. Allergic asthma is characterized by the activation of Th2 CD4+ T cells, which promotes an IgE-mediated response, activates mast cells, triggers an increase of eosinophils in the tissue and promotes bronchial hyperactivity. Upon allergen exposure and subsequent sensitization, a group of cytokines, IL-3, IL-4, IL-5, IL-9, IL-13 and GM-CSF, which are primarily Th2 type-cytokines, are released and may play a role in allergic asthma [7–9]. Recent studies have suggested that the mechanism of the Th2 response involves the epithelial production of TSLP (Thymic Stromal Lymphopoietin) during dendritic cell activation, which also leads to Th17 cell differentiation [10, 11]. IL-33 produced by endothelial and epithelial cells seems to potentiate the Th2 response, which worsens asthma [11]. In non-atopic asthma, skin tests are negative for specific allergens, and the serum levels of total IgE are normal or low [12, 13]. In adults, such a phenotype of asthma is characterized by a poor response to bronchodilators and thus requires the prolonged use of corticosteroids and presents a more rapid decline in PFT (Pulmonary Function Testing) parameters. Symptoms of non-atopic asthma are induced by nonspecific triggers, but the pathophysiological mechanisms are not yet fully understood [14]. With the recent discovery of iNKTs cells and innate lymphoid cells, researchers have suggested a possible role for these cells in both a mechanism for increasing asthma severity [15, 16] and a mechanism for the non-atopic asthma phenotype [15]. In addition, severe asthma phenotypes may be associated with the presence of Th17 cells, and the production of a Th17 profile (IL-17A, IL-17 F, IL-22, and IL-21) and IL-1β, which was shown to induce a Th17 profile and induces an increase in airway inflammation predominantly within neutrophil cells [17, 18]. According to the hygiene hypothesis, a reduction in exposure to variety of microorganisms, improvement of hygiene and sanitation, vaccines and the advent of widespread use of antibiotics has increased the prevalence of allergic diseases worldwide, linking the lack of microbial exposures in early childhood to increased susceptibility and the development of allergic diseases [4, 17]. An extension of the hygiene hypothesis, the “Old Friends” mechanism, suggests that urbanization over the last centuries has restricted human exposure to pathogens that are considered “old friends” of humanity, such as ancestral strains of *Mycobacterium tuberculosis* and *Helicobacter pylori*, intestinal helminthes and the Hepatitis A virus. This lack of exposure may have increased the prevalence of many allergic diseases in urban populations, including asthma [19, 20]. In this way, exposure to these “old friend” pathogens appears to be important to maturate immune cells and, more importantly, to mount a proper immune response and teach immune cells how to properly control inflammation [21]. The main mechanism whereby this can occur is through the activation of Treg cells [22] by pathogens such as viruses, bacteria and parasites [23]. There are twoTreg cell subtypes: FOXP3+ Treg cells and the Type 1 regulatory cells (Tr1). The first cell subtype expresses FOXP3 and is subdivided into thymus-derived Treg cells (tTreg) and peripheral Treg cells (pTregs). The second Tr1 cell subtype does not express the FOXP3 transcription factor to exert its functions [24]. The regulatory effects of FOXP3+ Treg cells are due to its repression of IL-2 production and its induction of CTLA-4 expression. In contrast, the activity of Tr1 cells are dependent on IL-10 production, regardless of the FOXP3 expression levels [25–27].

**Fig. 1:**
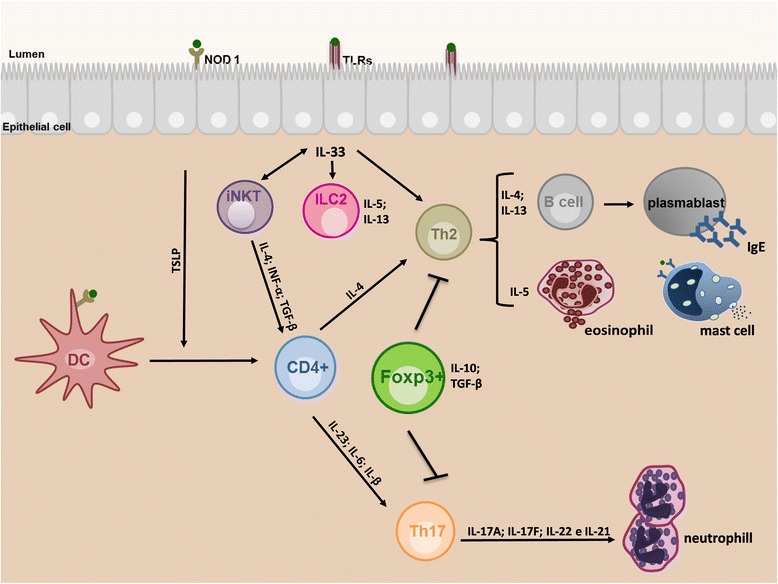
Mechanism of asthma development. The physiopathological mechanism of asthma involves three complex currently mechanisms: 1. The polarization of Th2 response with the production of cytokines such as IL-4, IL5 and IL-13, participation of sIgE, mast cell degranulation and predominance of eosinophils (classic atopic asthma); 2. Predominant participation of Th17 response, production of IL-17A; IL-17 F; IL-21 and IL-22 cytokines and the presence of neutrophils (probable mechanism of non-atopic asthma or increasing severity of atopic asthma); 3. Through the innate immunity activation where two main actions could be involved, the release of cytokines from epithelial cells, TSLP and IL-33, and the interaction between iNKTs and ILCs cells. The TSLP acts on the activation of dendritic cells and induction of Th2 response, and differentiation of T cells in Th17 profile. The IL-33 acts on the interaction between iNKTs and ILCs, but also acts enriching Th2-type cells. Evidence that suggests the FOXP3 transcription factor, which is constitutively expressed in CD4 + CD25 + Foxp3 + regulatory T cells (Treg) is critical for the maintenance of homeostasis and immune systemand alsoare responsible for the suppression of the Th2 and Th17 responses. DC = dendritic cells; sIgE = specificIgE; TLSP = thymic stromal lymphopoietin; iNKTs = invariant natural killer T; ILC2s = type 2innate lymphoid cells

Evidence suggests that the transcription factor FOXP3, which is constitutively expressed in CD4^+^CD25^+^Foxp3^+^ regulatory T cells (Tregs), are critical for the maintenance of immune system homeostasis and are responsible for the suppression of Th2 responses following exposure to allergens [28] (Fig. 1).

Several studies have shown that allergic patients, including asthmatics, have lower levels of Tregs in both the bronchoalveolar lavage and peripheral blood monocytes cells (PBMC) compared with healthy subjects [29, 30].

However, these associations remain unclear. Provoost et al. 2009 showed that the numbers of peripheral blood Treg-cells were similar in control subjects and asthmatic patients [31]. Other authors have shown that patients with atopic asthma have increased levels of Treg in peripheral blood compared with healthy individuals, but not non-atopic asthmatic individuals [4, 18].

Also, the FOXP3 levels in asthmatic patients are controversial. Several studies have shown that FOXP3 protein expression within Treg-cells is significantly decreased in asthmatic patients [4, 31, 32], which may result in failure of Treg cells to suppress Th proliferation and the production of cytokines observed in those patients [4, 30, 33]. However, in a recent study was described a higher FOXP3 expression in asthmatic patients than healthy individuals and the Treg cell–suppressive capacity was observed in both groups [18].

These divergence can be explained by a methodological differences between studies or because different Treg sub-types were used or because those studies were performed in the PBMC and thus represent a systemic response that may be influenced by the environment. Alternatively, the increased number of Treg cells in asthmatic patients, particularly patients with atopic asthma, may indicate a counter-regulatory mechanism that is yet not sufficient to control allergic inflammation.

Strategies to enhance the regulatory transcription factor FOXP3 have been used to treat or prevent allergic disease. The main approach to control allergy and asthma is corticosteroid therapy, either ingested or inhaled, both of which are associated with enhanced Foxp3+ expression and an increased suppressor function [34]. Recently, a novel therapeutic approach tested in mouse aimed to up-regulate FOXP3 expression in a time- and site-specific manner by administering an intra-tracheal instillation of plasmid that contains the mouse *Foxp3* gene. This approach led to an attenuation of airway inflammation by reducing the Th2 immune response [35, 36]. Thus, identifying genetically susceptible individuals in association with the development of treatment strategies would be of great relevance for managing allergic asthma.

### Structure and function of FOXP3

Forkhead box (FOX) proteins constitute an evolutionarily conserved family of transcription factors with a central role not only during development but also in the adult organism [37]. This protein is expressed by T cells and primarily functions to promote the differentiation of TCD4 + CD25+ cells and stimulate their suppressive activity [38, 39]. The term, “winged helix”, which is used to describe its structure, derives from a helix–turn–helix core of three α-helices that are flanked by two loops or “wings”. There is an ~110-amino-acid DNA binding forkhead domain, which is highly conserved. Thus, there is a defined 3D structure and mode of DNA recognition for this forkhead family of transcription factors [38].

**Fig. 2:**
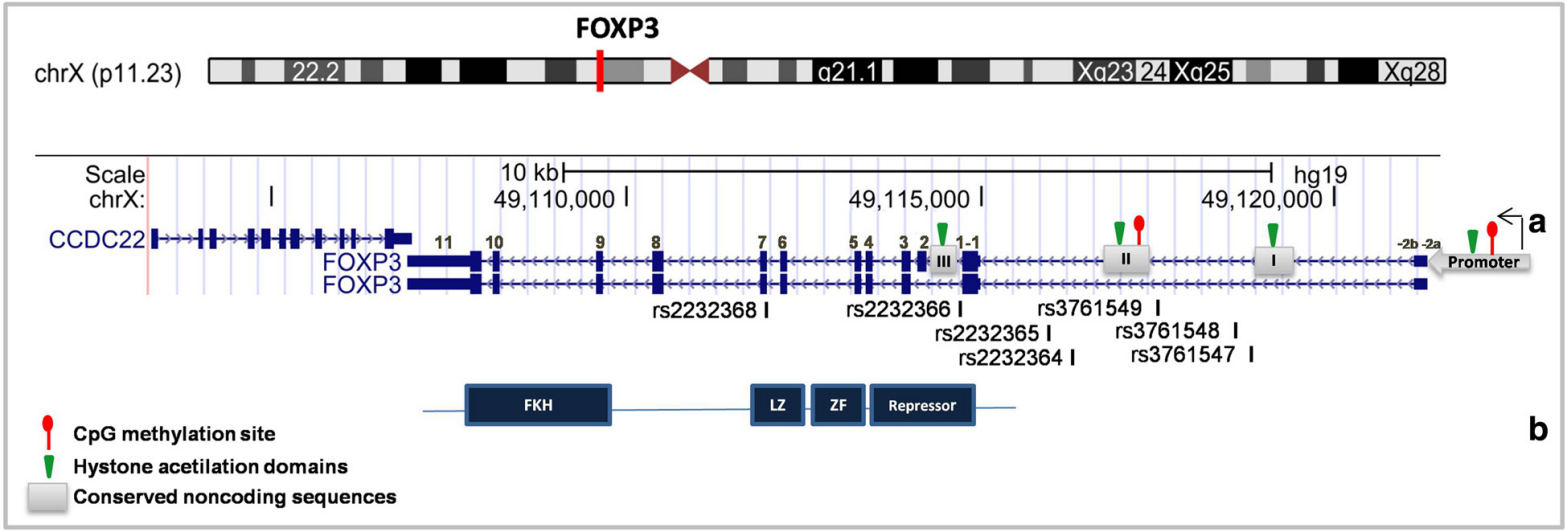
Schematic diagram of the *FOXP3* gene (**a**), protein (**b**) and X Chromosome. The figure shows two isoforms of the gene (with difference in exon 2) and some SNPs cited in the text. ZF: zinc finger domain, LZ: leucine zipper domain, and FKH fork-head domain.. The chromosome and gene schematic diagram was modified from NCBI Reference Sequence (RefSeq)

This protein contains 431 amino acids with four functional domains, as shown in Fig. 2. Although previous research has shown that the fragment responsible for NFAT inhibition is in the N-terminal, another study demonstrated that removal of the FOXP3 C-terminal prevents NFAT binding [40].

The expression of this transcription factor in T cells is related to the proliferation of regulatory T cells, which exert their suppressive activity on T helper cells to regulate the inflammatory response [41].

The role of FOXP3 in Treg cells has been demonstrated in studies that suppress the function of this gene and through adoptive transfer experiments. Several authors have shown that after the knockout of FOXP3, Treg cells lose their suppressive activity and start to produce IL-2 and Th1 cytokines. Similar to these findings, the adoptive transfer of FOXP3 retrieves the regulatory function of T cells and suppresses lymphoproliferative activity [42–44].

The importance of FOXP3 for immune system function was demonstrated in scurfy mice with lymphoproliferative disease and an X-linked condition that was caused by a mutation in FOXP3 that deletes the C-terminal domain. These animals are deficient in the production of regulatory T cells and present with clinical symptoms, such as exfoliative dermatitis, weight loss, presence of auto-antibodies, lymphadenopathy and lymphocytic infiltrates, which lead to animal death in approximately 3 weeks [45].

Many studies have investigated the specific domains and, consequently, the function of FOXP3. Mutations in the forkhead domain at amino acid positions 415 and 416 abolished nuclear migration of FOXP3. Mutations in the leucine zipper domain cause a loss of dimerization and thus reduce FOXP3 binding to promoter regions [46, 47].

FOXP3 is critically important for regulating the immune system and can suppress NFAT function, thereby inhibiting NFAT complex formation with AP-1 and inflammatory pathway activation. NFAT is bound in its promoter region by FOXP3, which primarily serves to down-regulate IL-2 and IL-4 and to up-regulate CTL-4 and CD25 [48]. FOXP family members can form dimmers and activate transcription [49]. Members of this subfamily include FOXP1, FOXP2, FOXP3 AND FOXP4. FOXP1, FOXP2 and FOXP4 are expressed in gut, brain and lung and appear to have a role in embryogenesis that is maintained in adults [49]. FOXP1 and FOXP2 activity is found in the immune system [49, 50]. The co-expression and heterodimer formation of FOXP1/FOXP3 has been reported. FOXP1 is present in both CD4 + CD25+ and CD4 + CD25- T cells, whereas FOXP3 is expressed only in CD4 + CD25+ T cells. In mice with IPEX syndrome, the depletion of E251 impaired the heterodimerization of FOXP3 with FOXP1, thus suggesting a role for such heterodimerization in suppressive immune activity [50].

### The role of *FOXP3* polymorphism in asthma and allergic diseases

The human *FOXP3* gene is located on the X-chromosome (Xp11.23), is 1296 bp in size, and contains 11 coding exons and 3 noncoding exons. The *FOXP3* gene belongs to a family of molecular complexes that are ~600 kd all together and includes histone deacetylases and acetyltransferases, as well as other transcription factors such as RUNX1 and NFAT1 [48, 51, 52]. Fig. 2 shows a schematic diagram of the FOXP3 gene.

As observed in Fig. 2, two upstream 5’ noncoding exons (−2a and−2b) are separated by 640 base pairs and are linked at the second noncoding exon (−1). The -2b and −1 exons are separated by five hundred base pairs and have several cis-regulated elements [53, 54]. The *FOXP3* gene has more than a hundred single nucleotide polymorphisms (SNPs), nearly twenty of which have been studied for association with different diseases [55–58].

SNPs are the most common variations in the genome and are responsible for individual phenotypic differences. The coding sequences of genes are often conserved, but the presence of SNPs or genetic mutations may be related to the susceptibility to complex diseases. The role of host genetic factors in the etiology of complex diseases is generally studied using Genome-Wide Association Studies (GWAS) or Candidate Gene Studies. Many GWAS have investigated the influence of genetic polymorphisms on the development allergic diseases [59–61], but few studies have included the X chromosome because it is difficult to analyze [62]. The X chromosome contains more than 300,000 SNPs on 2300 genes, almost all of which encode proteins, such as *FOXP3* [63, 64]. GWAS of asthma have successful identified genetic susceptibility; however, little information about the X chromosome has been reported, and no information about the association of FOXP3 SNPs on allergic diseases has emerged [59–61]. A notable exception is the Moffatt et al. 2010 study, which analyzed the X chromosome in populations with predominant European ancestry but reported no statistically significant association signals [65]. However, it is important to note that X chromosomal variants are often underrepresented in genotyping platforms compared with autosomal chromosomes [66]. The fact is that the analysis of SNPs within the X chromosome can provide important information regarding genetic factors associated with diseases and should not be neglected. GWAS are the most powerful approach to identify the genetic risk for asthma, but candidate gene studies are the most common, and the results of these studies on *FOXP3* are discussed here.

Recently, the *FOXP3* gene has been investigated in association studies for many diseases [55–57]. Mutations in this gene may be associated with the development of Immune dysregulationpolyendocrinopathy and enteropathy X-linked (IPEX) syndrome, a rare and fatal pediatric condition. Bennett et al. 2001 identified a mutation in the *FOXP3* gene in patients with IPEX who exhibited aggressive autoimmune features [45]. This finding suggests that the genetic variations in *FOXP3* gene may be associated with T cell dysfunction. Thus, host genetic factors that affect *FOXP3* can determine differences in susceptibility to allergic diseases such as asthma.

Over the last few years, polymorphisms in this gene have been evaluated in association studies for several allergies [67–69], but few studies in asthma were conducted. Therefore, we here discuss the major findings concerning the FOXP3 gene in association studies for asthma and other allergic conditions. Table 1 presents all of the SNPs in the FOXP3 gene that have been published to date for asthma and allergic diseases, including the sample size.

**Table 1:** *FOXP3* SNPs investigated for association with asthma and allergy

SNP	Genomic Position	Alleles	Function	Diseases	N (cases/controls)	Country	Reference
rs3761548	49,261,784	A/C	Intron	Atopy	3062*	The Netherlands	Bottema, 2009
				Allergic rhinitis	395	Hungary	Fodor, 2010
					(178/217)		
				Asthma	3062*	The Netherlands	Bottema, 2010
				Allergic rhinitis	384	China	Zhang, 2009
					(193/191)		
				Allergic rhinitis	318	Iran	Hassannia, 2011
					(153/165)		
				Allergic rhinitis	708	China	Zhang, 2012
					(378/330)		
rs2232365	49,259,429	A/G	Intron]	Allergic rhinitis	384	China	Zhang, 2009
					(193/191)		
				Allergic rhinitis	708	China	Zhang, 2012
					(378/330)		
				Allergic rhinitis	318	Iran	Hassannia, 2011
					(153/165)		
rs6609857	49,245,158	C/T	3’ UTR	Asthma	3062*	The Netherlands	Bottema, 2010
				Atopy	3062*	The Netherlands	Bottema, 2009
rs2232368	49,255,822	A/G	Intron	Allergic rhinitis	384	China	Zhang, 2009
					(193/191)		
rs2232366	49,258,209	G/T	Intron	Allergic rhinitis	384	China	Zhang, 2009
					(193/191)		
rs2232364	49,259,888	A/C/G/T	Intron	Allergic rhinitis	384	China	Zhang, 2009
					(193/191)		
rs3761549	49,260,888	C/T	Intron	Atopy	3062*	The Netherlands	Bottema, 2009
rs3761547	49,262,004	A/G	Intron	Allergic rhinitis	384	China	Zhang, 2009
					(193/191)		
rs2869211	49,264,409	A/T	Intron	Allergic rhinitis	384	China	Zhang, 2009
					(193/191)		

Cases and controls were not shown for all studies (*) because some studies use different phenotypes and analyze sex and age separately

### rs3761548

The rs3761548 is located in the intronic region of the *FOXP3* gene. It is the most studied SNP for *FOXP3* and has been associated with several diseases, including many allergic conditions.

Bottema et al. 2009 studied the association of this SNP with atopy and observed no significant association with IgE levels; however, an association was found to food sensitivity to egg allergens (OR: 0.5; 95 % CI 0.3–1.0) [67]. In addition, another study reported significant interaction (*p* < 0.01) between SNPs in *FOXP3*-*IL2R* genes and IgE for eggs and asthma [70].

In association studies with allergic rhinitis (AR), Hassannia 2011 reported that the AC genotype for this rs3761548allele was protective for AR in females (OR, 0.16; 95 % CI 0.05–0.5) but that the C allele was protective (OR: 0.47; 95 % CI 0.22–0.99) for AR in males [71]. However, a study conducted in Hungary found protection *p* < 0.05 for allergic rhinitis only in females who carried the AA genotype [68]. A similar finding was reported in another study that found a positive association (OR: 3.12; 95 % CI 1.21–8.04) between the heterozygous genotype and AR [69]. In a haplotype analysis, Zhang 2012 found that the diplotype rs3761548–rs4824747 with “AG” was associated (OR: 1.75; 95 % CI 1.05–2.92) with a significantly increased risk of AR [72]. In addition to these findings, Hassannia et al. 2011 reported that women with genotype AC and CC showed reduced levels of total serum IgE. In men, the presence of the C allele was associated with a reduction in the total serum IgE levels [71].

Thus, this polymorphism appears to contribute to the risk of allergic disease, but further studies are needed to determine its effects on asthma.

### rs2232365

This SNP is located in the intronic region of the *FOXP3* gene. Although it has been included in several studies of different diseases, few studies on AR have included it. No association of this SNP was found with either AR [69, 71, 72] or the levels of IgE and peripheral blood eosinophil [71]. The same results were observed for association using haplotype analysis [72]. These data suggest that rs2232365 likely does not play an important role in AR, but its roles in other allergic diseases, such as asthma and atopy remain unclear.

### rs6609857

The rs6609857SNP is located near the 3' UTR region of the *FOXP3* gene, and although it is characterized as part of the *FOXP3* gene, its genomic physical position is located in the *CCDC22*gene (coiled-coil domain containing 22). This marker has been investigated in the context of asthma, allergy and IgE, but none of these studies have implicated rs6609857 as a risk factor for these outcomes [67].

### Other SNPs

The SNPs rs2294019 and rs5906761 were associated (OR: 3.9; 95 % CI 1.2–12.5 and OR: 4.1; 95 % CI 1.1–15.4, respectively) with a risk to egg sensitivity only in females [67]. The heterozygote genotype for rs3761547 was a risk factor for allergic rhinitis, and this association was reproduced in gene-gene interaction analysis with rs3761548 [69, 72].

Taken together, these results all show that polymorphism in *FOXP3* gene is associated with some allergic disease, but its contribution to asthma has been poorly studied. Moreover, there is heterogeneity in the sample size and population, which makes it difficult to compare the different studies. Thus, more studies are needed to evaluate the role of *FOXP3* polymorphisms in allergic diseases.

### Epigenetic regulation of FOXP3 in asthma

The constitutive expression of FOXP3 is required for the immunosuppressive function of Treg cells. In addition to the activity of trans-acting factors, epigenetic modifications play a central role in maintaining the stability of Treg cells. Epigenetics refers to changes in gene expression that are not caused by changes in the DNA sequence. Epigenetic mechanisms include DNA methylation and histone modification. DNA methylation occurs predominantly at CpG nucleotides and is catalyzed by DNA methyltransferases (DNMts). DNA methylation can inhibit gene expression directly by precluding the binding of specific transcription factors in promoter region of genes, or indirectly by promoting the recruitment of methyl-CpG-binding domain (MBD) proteins and their associated histone-modifying and chromatin-remodeling complexes [73]. Histones are protein constituents of nucleosomes that are subjected to different post-translational modifications in their N-terminal tails, including acetylation, methylation, phosphorylation, ubiquitination, SUMOylation and ADPribosylation [74]. Histone acetylation is catalyzed by histone acetyltransferase, and acetyl groups are removed by histone deacetylases (HDACs). Whereas histone acetylation results in open chromatin that permits recruitment of transcriptional machinery, deacetylation catalyzed by HDACs leads to the formation of closely compact chromatin that inhibits transcription.

Four *FOXP3* regions are susceptible to epigenetic modification in conserved noncoding sequences of DNA (CNS). These regions are the promoter region, enhancers [1, 2] and the pioneer element region (Fig. 2). In the promoter region, CpG motifs are partially methylated in CD4+ *naive* cells and demethylated in regulatory T-cells. The first enhancer region, which is formed by CNS-1, is susceptible to histone acetylation, but has no CpG motifs. This region is rich in linking sites for NFAT and Smad3 [75]. The second enhancer region is formed by CNS-2 and is known as the Treg-cell-specific demethylation region (TSDR) [76, 77]. The CpG motifs in this region are methylated in conventional T cells and demethylated in natural Tregs. Additionally, histones near this region are acetylated in thymus-derived Treg cells [78]. The pioneer element region in *FOXP3* is responsible for regulating the size, composition and stability of T regulatory cell family members [79]. Specifically, the CNS-3 enhances the frequency of Treg cell generation in both, thymus and in the periphery [79]. Chromatin modification marks at this site are permissive in Treg. In addition, the mono- and di-methylation patterns observed in Treg-precursors are absent in CNS-1 and CNS-2, which allows transcription factors to bind preferentially to this area instead of binding to CNS-1 or CNS-2 [78].

Several lines of evidence show that epigenetic changes in the *FOXP3* locus of Treg cells influence the asthma phenotypes. A summary of these studies, including their sample sizes, is shown in Table 2.

**Table 2:** *FOXP3* epigenetic studies investigated for association with asthma and allergy

Author(s)	Year	n (cases/controls)	Epigenetic marker analyzed	Cell population analysed
Nadeau et al.	2010	32 (16/16)	CpG methylation	Treg cells (CD4^+^CD25^hi^CD127^lo^) and effector T (Teff) cells (CD4^+^CD25^lo/neg^)
Brunst et al.	2013	71 (15/56)	CpG methylation	Buccal cells in saliva
Runyon et al.	2012	42 (21/21)	CpG methylation	Treg cells (CD4^+^ CD25^hi^CD127^lo/neg^) and Teff CD4^+^CD25^neg^
Kohli et al.	2012	102 (37/65)	CpG methylation	Treg cells (CD4^+^ CD25^hi^CD127^lo^) and Teff CD4^+^CD25^neg^
Lluis et al.	2014	43	CpG methylation	Whole blood
Michel et al.	2013	95 (45/50)	CpG methylation	Cord blood and whole blood

Nadeau et al. reported that among individuals who were exposed to both high and low levels of environmental pollutants, FOXP3 mRNA expression and Treg cell function were reduced in children with asthma compared to children without asthma. Accordingly, the methylation of CpG islands located in the promoter and in intronic regions of FOXP3 in Treg cells was higher in asthmatics relative to children without asthma, with a greater effect being observed in children who were exposed to high levels of pollution. The percentage of methylated CpG motifs in asthmatic and non-asthmatic individuals was ~60 % and ~45 %, *p* < 0.01, respectively [80]. The hypermethylation of FOXP3 in buccal cells was associated with a risk of persistent asthma and wheezing in childhood (OR: 3.05; 95 % CI 1.54–6.05). In addition, a positive correlation was observed between *FOXP3* methylation and exposure to chronic diesel exhaust particles (DEP) (4.01 %, 95 % CI 1.83–6.18 %; increase in FOXP3 methylation per interquartile range increase in estimated DEP exposure) [81]. A study of monozygotic twins (MZT) pairs that were discordant for asthma found a decrease in FOXP3 protein expression and impaired Treg function in the asthmatic twin, both of which were associated with increased levels of CpG methylation within the FOXP3 locus. CpG sites within FOXP3 were almost six times more methylated in the asthmatic MZT *vs* the non-asthmatic MZT, *p* < 0.001. Furthermore, these effects were increased by current exposure to second-hand smoke (SHS) [82]. In addition, SHS and air pollution exposure, which have been associated with an increased prevalence and severity of asthma, were positively associated with hypermethylation and the decreased expression of FOXP3 in Tregs. The mean % CpG methylation of FOXP3 among SHS-exposed *vs* non-SHS-exposed was 74.60 % *vs* 54.44 %, respectively, *p* < 0.05, and the mean transcription levels of FOXP3 among SHS-exposed and non-SHS-exposed were 0.75 and 3.29, respectively, *p* < 0.05 [83]. These results suggest that exposure to certain environmental factors, such as pollutants, may induce epigenetic modifications in the FOXP3 locus with a consequently increased risk of asthma.

Lluis et al. showed that farm milk consumption was inversely associated with doctor-diagnosed asthma at age 4 years (OR: 0.26; 95 % CI 0.08–0.88) and that FOXP3 demethylation at the TSDR region was consistently higher, although no significant, in whole blood of children who had consumed farm milk (median differences for all CpGs, *p* = 0.08). In addition, protection against asthma by farm milk exposure was partially mediated by Treg cells [84]. However, no difference in the *FOXP3* methylation status was observed in children with farm exposure in general compared to those without exposure [85]. This latter result suggests that the effect of farm environment on the epigenetic modification of *FOXP3* is specific to the type of exposures and may not be detected, depending on the exposure assessment. Interestingly, a recent study showed that patients with atopic asthma had a trend-wise higher average level of histone H3 acetylation in the *FOXP3* promoter region compared with healthy controls, although this difference was not statistically significant (*p* = 0.07, *n* = 26, for the mean difference in H3 acetylation between atopic asthma cases and controls) [86]. Because histone acetylation is associated with increased gene activity, an increased number of Treg cells would be expected in patients with atopic asthma, which was reported recently in a study conducted in this same population [18]. These results suggest that the status of Treg cells may differ according to the asthma phenotype considered (allergic or non-allergic).

Finally, the administration of 5-azacytidine (Aza), a DNA methyltransferase inhibitor, to chicken ovalbumin (OVA)-sensitized mice decreased airway hyperreactivity, pulmonary eosinophilia, the levels of OVA-specific IgG1 and IgE in serum, and secretion of Th2 cytokines from OVA-stimulated splenocytes in a dose-dependent manner. Furthermore, the number of Treg cells was remarkably increased in Aza-treated mice compared with sensitized control mice [87]. These data indicate that epigenetic regulation of Treg might contribute to the modulation of asthma-induced airway inflammation, which opens the possibility for treating allergic asthma and other allergic diseases by using epigenetic therapeutic agents.

## Conclusions

Polymorphisms in the FOXP3 gene have been associated with some allergic diseases but the contribution of these polymorphisms to asthma development has been poorly studied. Several lines of evidence point to the involvement of epigenetic changes in the *FOXP3* locus of Treg cells in asthma phenotypes. Further investigation will be important to clarify the role of *FOXP3* polymorphisms and epigenetics mechanisms on the risk of asthma and other allergic diseases. Furthermore, genome-wide analyses of epigenetic markers in Treg cells are needed to enrich our ability to develop epigenetic therapeutic approaches to asthma and allergies.
